# Effect of Mass Stool Examination and Mass Treatment For Decreasing Intestinal Helminth and Protozoan Infection Rates in Bolivian Children: A Cross-Sectional Study

**DOI:** 10.1371/journal.pntd.0005147

**Published:** 2016-12-06

**Authors:** Takao Asai, Claudia Còrdova Vidal, Wilma Strauss, Toshikazu Ikoma, Kazuo Endoh, Masaharu Yamamoto

**Affiliations:** 1 Faculty of Medical Technology, Niigata University of Health and Welfare, Niigata, Japan; 2 Laboratorio de analisis clinicos Alfa & Omega, La Paz, Bolivia; 3 Cátedra de Parasitología, Facultad de Farmacia y Bioquímica, Universidad Mayor de San Andrés, La Paz, Bolivia; 4 Hokuriku University, Kanazawa, Ishikawa, Japan; 5 Faculty of Health Sciences, Niigata University of Health and Welfare, Niigata, Japan; Walter and Eliza Hall Institute, AUSTRALIA

## Abstract

Bolivia is one of the countries with a high intestinal helminth and protozoan infection rate. Despite the high prevalence of the parasitic infection, nationwide preventive measures for Bolivian children have not yet been implemented. We evaluated the effect of mass stool examination and treatment as a strategy for decreasing the infection rate. This study was conducted between 2013 and 2015 in children aged 2–18 years. A total of 2,033 stool samples (575 in 2013, 815 in 2014 and 642 in 2015) were collected and examined using the formalin-ether medical sedimentation method. As an anthelminthic medicine, nitazoxanide was given to all infected children within 2 months post-examination, each year. The effect of mass stool examination and treatment was evaluated based on the changes in the overall or individual parasitic infection rates during the study period. The overall parasitic infection rate decreased significantly from 65.2% in 2013 to 43.0% in 2015; a 22.2 percentage point decrease (P<0.001). Protozoan infection accounted for a large portion of the parasitic infections, in the following rates: 62.4% in 2013, 49.3% in 2014, and 41.0% in 2015. The rate of the most common helminth infection, *Hymenolepis nana*, decreased significantly from 9.0% in 2013 to 6.4% in 2014 to 3.4% in 2015 (P<0.001). Prevalence of the most common pathogenic protozoan infection, *Entamoeba histolytica*, decreased significantly from 19.0% in 2013 to 3.0% in 2015 (P<0.001). Conversely, the rate of *Giardia intestinalis* increased significantly from 16.5% in 2013 to 21.2% in 2015 (P<0.01). Mass stool examination and treatment for intestinal helminth and protozoan infections was effective for decreasing the overall parasitic infection rate in the study population, excluding *Giardia intestinalis*. Further studies on the long-term effect of mass stool examination and treatment for decreasing all intestinal parasitic infection rates in Bolivian children are needed.

## Author Summary

Intestinal helminth and protozoan (parasitic) infections are found mainly in areas with warm, humid climates and in many developing countries. Bolivia currently has high parasite infection rates, including intestinal helminth and protozoan infections. Despite the high prevalence of parasitic infections, nationwide preventive measures for Bolivian children have not yet been implemented. Soil-transmitted helminth infection rates in Japan decreased sharply through practice of health education, mass stool examination and treatment. We investigated the effects of mass stool examination and treatment on parasitic infection rates in Bolivian children. Mass stool examination was performed using the formalin-ether medical sedimentation method between 2013 and 2015, and all infected children were administrated anthelminthic medicine within 2 months post-examination. During the study period, the overall infection rate decreased significantly, but *Giardia intestinalis* infection rate increased. This may have been related to the interval between treatment and examination. To decrease infection rates for all parasites, the treatment should be performed once or twice a year. Our results suggest that a program of mass stool examination and treatment could effectively reduce the overall parasite-infection rate in Bolivian children.

## Introduction

Intestinal parasitic infections by organisms including helminths and protozoa are among the most prevalent infections in the world [1]. According to data provided by the United States Centers for Disease Control and Prevention (CDC) in 2013, a large part of the world’s population is infected with soil-transmitted helminths such as *Ascaris lumbricoides* (*A*. *lumbricoides*), *Trichuris trichiura* (*T*. *trichiura*), and *Necator americanus* or *Ancylostoma duodenale* (Hookworm) [2]. The number of people infected with *A*. *lumbricoides* was estimated to be 807 million to 1,121 million, 604 million to 795 million were estimated to be infected with *T*. *trichiura*, and 576 million to 740 million were estimated to be infected with hookworm. Regarding endemic areas, the CDC reported that the highest rates of parasitic infection are found in warm and humid areas of Latin America, Africa, and Asia [3]. These infections are uncommon in Australia, Canada, Europe, Japan, New Zealand and the United States [4].

Intestinal helminth infection rates, especially those caused by soil-transmitted helminths, are high in children aged 1–14 years [5]. For this reason, the WHO recommends periodic treatment with anthelminthic medicines, without previous stool examination, for all at-risk people, including preschool-aged children, school-aged children and childbearing-aged women who live in endemic areas. This treatment is recommended once a year when the prevalence of the infections in the community is over 20% and twice a year when the prevalence exceeds 50% [6]. Intestinal protozoan infections are also widespread in countries or in areas where sanitation and hygiene provisions are inadequate [7]. These infections are predominantly transmitted through the fecal-oral route. Intestinal protozoan infections are common in school-aged children in developing countries, but even in the United States, the most common intestinal parasitic disease affecting humans has been reported to be *Giardia* infection [8]. According to the surveillance data on *Giardia* infection conducted in the United States during 2011 and 2012, the largest number of cases and the highest average annual incidence rate have been reported to be among young children aged 1–9 years [9]. These findings suggest that the continued and systematic effort for preventive measures against intestinal helminth and protozoan infections in children is needed.

The Plurinational State of Bolivia (Bolivia) has a high prevalence of intestinal parasitic infection. To our knowledge, there are no data on the nationwide prevalence of parasitic infections in Bolivia. Previous studies have shown the following overall parasitic infection rates in Bolivian children: 77.2% (helminths, 76.1%; protozoa, 56.5%) [10], 65.1% (helminths, 30.6%; protozoa, 20.8%, helminths and protozoa, 13.7%) [11], 69.0% (helminths, 4.1%; protozoa, 61.6%; helminths and protozoa, 3.3%) [12] in lowland Bolivia, and 18.0% (helminths only) [13] or 43.8% (helminths, 1.8%; protozoa, 39.1%; helminths and protozoa, 2.9%) [14] in a high altitude area of La Paz, the capital of Bolivia. These findings demonstrate intestinal parasitic infection rates are higher in lowland Bolivia than La Paz, a high altitude area. Protozoan infection was shown to be high in both lowlands and high altitude areas. Therefore, we should give the same attention as helminth infection to protozoan infections. Based on these infection rates and the WHO’s strategy for control of soil-transmitted helminth infections, Bolivian children should be treated with anthelminthic medicines once or twice a year. However, to our knowledge, the children living in our study area have not yet been treated with anthelminthic medicines, and no research on the effect of treatment with anthelminthic medicines has been published.

At the beginning of the 20^th^ century, Japan had high soil-transmitted helminth infection rates. According to the annual report of the Tokyo Health Service Association, the overall infection rate among citizens of Tokyo was 61.4% in 1950, and thereafter the infection rate fell as a result of health education, mass stool examination, and mass treatment. Between 1967 and 2000, the rate was reported to be less than 1%, and no mass stool examination for soil-transmitted helminths has been conducted since 2002 [15]. Among the factors reducing the parasitic infection in Japan were mass stool examination and treatment. One of the factors that contributed to the success of parasite control activity was that it was performed throughout Japan through a tripartite collaboration between government officers, scientists, and the private sector for parasite control (voluntary organizations, Japan Association of Parasite Control; private organization and laboratories, the branch offices of the Japan Association of Parasite Control) [16]. This activity then spread to other Asian countries and led on to decrease soil-transmitted helminth infection rates in Korea [17], Taiwan [18], and Indonesia [19]. If parasite control activity was carried out through a tripartite collaboration in Bolivia, the intestinal helminth and protozoan infection rates would reduce considerably.

We therefore conducted this study to evaluate the effect of mass stool examination and treatment as measures to decrease intestinal parasitic infection rates in Bolivian children. We hypothesized that the parasitic infection rate in Bolivians would be reduced sharply by mass stool examination and treatment.

## Methods

### Study region

This study was conducted in the Mecapaca area (16°43’0” S, 67°59’0” W) located 30 km south of La Paz, which is the capital of Bolivia and the administrative seat of Bolivia’s government. This area has a total land area of 533 km^2^ and is at an altitude of approximately 2,800 meters. The population was 17,925 as of 2012 [20]. The average temperatures in this area range between highs of 14–17°C and lows of -4–4°C [21].

### Subjects

This study was conducted with extensive cooperation from a local school comprising kindergarten, elementary, junior high, and high school students. There were approximately 1,000 children aged 1–19 years old in each year in the school.

### Stool sample collection, mass stool examination and treatment

A total of 2,033 stool samples were collected from the study population: 575 in 2013, 816 in 2014 and 642 in 2015. The samples were examined using the medical general laboratory method (formalin-ether medical sedimentation method) [22]. The sediment was examined using a light microscope. Iodine solution was used to identify protozoa cysts, which were dyed pale yellow or brown. The classification of parasites was carried out based on outline images from a specialist book [23]. For quality control, all the samples were examined by both Bolivian and Japanese researchers and the results were consistent.

First, to provide baseline data, we conducted a stool examination for 575 children in September 2013. The data from 2013 were used as a baseline for the study because this study was to do a cross-sectional analysis of baseline data. After the examination, nitazoxanide (as an anthelminthic medicine), was administrated to all infected children between October and November 2013. To clarify the effect of nitazoxanide administration, stool examination was carried out for all children in March 2014. This examination was carried out between 4 and 5 months post-administration and all infected children were given nitazoxanide between April and May 2014. A further stool examination was carried out for all the children at 16–17 months post-2014 administration. Anthelminthic medicine was administered for infected children between October and November 2015.

Nitazoxanide was administrated at a dose of 500 mg tablet with water or food for the children aged 12 years and older; 7.5 mL syrup for the children aged 11 years and younger, once a day.

### Ethics Statement

The study procedure was reviewed and approved by the Ethics Committee of the Niigata University of Health and Welfare (No. 17298–120125), and was conducted according to the principles expressed in the 1964 Helsinki Declaration. Prior to the start of the 2013–2015 study, a Bolivian researcher provided information on the purpose and procedure of the study to parent(s) or guardian(s) of children; risks and benefits of the examination and treatment; risks and benefits of not undergoing examination and treatment. Subsequently, the researcher requested oral informed consent from parent(s) or guardian(s) on behalf of their children. The container with the serial number for stool sample collection was given to parent(s) or guardian(s) of children who agreed to participate in the study. We considered that the parent(s) or guardian(s) gave the informed consent on behalf of the children when they submitted their children’s fecal samples.

### Statistical analysis

The effect of mass stool examination and treatment was evaluated by the changes in parasitic infection rates during the study period. No significant gender differences in the overall infection rate were shown in previous studies conducted in Bolivia [12–14] or in other countries [24,25], so we did not assess difference in infection rate between boys and girls. All statistical analyses were performed using Stata Data Analysis and Statistical Software (STATA 14; StataCorp, College Station, TX, USA). The changes in parasitic infection rates between 2013 and 2015 were analyzed using the Cochran–Armitage trend test [26–28]. P values of less than 0.05 (two-tailed) were considered to indicate statistical significance.

## Results

Table 1 shows the changes in intestinal parasitic infection rates during the study period. The overall parasitic infection rate was 65.2% in the first year. Of the 575 children who submitted stool samples in 2013, 16 (2.8%) had helminth infection, 316 (54.9%) had protozoan infection, and 43 (7.5%) had both infections. After treatment with nitazoxanide in all infected children, the rates decreased to 51.6% in 2014 and 43.0% in 2015. The overall infection rate decreased significantly from 65.2% in 2013 to 43.0% in 2015, a 22.2 percentage point decrease (P<0.001). No significant difference was found in the rate of children with only helminth infection during the study period. Protozoan infection (protozoan and combined helminth and protozoan infections) accounted for a large portion of the parasitic infections, in the following rates: 62.4% in 2013, 49.3% in 2014, and 41.0% in 2015.

**Table 1:** Changes in intestinal parasitic infection rates of Bolivian children in the study in 2013, 2014, and 2015, by parasite type and year.

	2013 (n = 575)	2014 (n = 816)	2015 (n = 642)	P value
	No. (%)	No. (%)	No. (%)	
Helminths Protozoa Helminths and Protozoa	16 (2.8) 316 (54.9) 43 (7.5)	19 (2.3) 360 (44.1) 42 (5.2)	13 (2.0) 251 (39.1) 12 (1.9)	0.39 <0.001 <0.001
Total	375 (65.2)	421 (51.6)	276 (43.0)	<0.001

Changes were analyzed using the Cochran–Armitage trend test.

Table 2 shows the changes in the number of helminth or protozoa species found in Bolivian children during the study period. Single-species infection was the most common form in the children—34.6% in 2013, 37.0% in 2014, and 31.0% in 2015—but did not change significantly by year (P = 0.16). The proportion of children infected with two to four species of parasite decreased significantly (P<0.001). The rates for two-species infection decreased from 19.1% in 2013 to 11.1% in 2015, for three-species infection decreased from 8.3% in 2013 to 0.9% in 2015, and for four-species infection decreased from 3.0% in 2013 to 0% in 2015.

**Table 2:** Changes in the number of helminth or protozoa species found in Bolivian children between 2013 and 2015.

	2013 (n = 575)	2014 (n = 816)	2015 (n = 642)	P value
	No. (%)	No. (%)	No. (%)	
0 species infection 1 species infection 2 species infection 3 species infection 4 species infection 5 species infection	200 (34.8) 199 (34.6) 110 (19.1) 48 (8.3) 17 (3.0) 1 (0.2)	395 (48.4) 302 (37.0) 98 (12.0) 17 (2.1) 3 (0.4) 1 (0.1)	366 (57.0) 199 (31.0) 71 (11.1) 6 (0.9) 0 (0) 0 (0)	<0.001 0.16 <0.001 <0.001 <0.001 0.33

Table 3 shows the changes in helminth and protozoan infection rates in Bolivian children during the study period. We found the following six helminths: *Hymenolepis nana* (*H*. *nana*), *A*. *lumbricoides*, *T*. *trichiura*, Hookworm, *Taenia saginata/solium*, and *Enterobius vermicularis*. The rate of the most common helminth infection, *H*. *nana*, decreased significantly from 9.0% in 2013 to 6.5% in 2014 to 3.4% in 2015 (P<0.001). *T*. *trichiura* was detected in three children in 2013, but in none in 2014 and 2015 the infection rate showed a significant decrease during the study period (P = 0.02). No significant changes occurred in the infection rates of *A*. *lumbricoides*, Hookworm, *Taenia saginata/solium*, and *Enterobius vermicularis* during the study period.

**Table 3:** Changes in helminth and protozoan infection rates of Bolivian children in the study in 2013, 2014, and 2015, by parasite species and year.

	2013 (n = 575)	2014 (n = 816)	2015 (n = 642)	P value
	No. (%)	No. (%)	No. (%)	
Helminths * Hymenolepis nana* * Ascaris lumbricoides* * Trichuris trichiura* * *Hookworm * Taenia saginata/solium* * Enterobius vermicularis*	52 (9.0) 6 (1.0) 3 (0.5) 1 (0.2) 1 (0.2) 0 (0)	53 (6.5) 4 (0.5) 0 (0) 0 (0) 0 (0) 4 (0.5)	22 (3.4) 2 (0.3) 0 (0) 0 (0) 0 (0) 1 (0.2)	<0.001 0.10 0.02 0.19 0.19 0.65
Protozoa * *Pathogenic protozoa * Entamoeba histolytica* * Giardia intestinalis* * *Nonpathogenic protozoa * Entamoeba coli* * Endolimax nana* * Chilomastix mesnili* * Iodamoeba buetschlii*	109 (19.0) 95 (16.5) 267 (46.4) 75 (13.0) 26 (4.5) 2 (0.3)	63 (7.7) 58 (7.1) 336 (41.2) 14 (1.7) 14 (1.7) 20 (2.4)	19 (3.0) 136 (21.2) 164 (25.5) 2 (0.3) 2 (0.3) 11 (1.7)	<0.001 0.01 <0.001 <0.001 <0.001 0.08

Hookworm: *Necator americanus* or *Ancylostoma duodenale*.

In addition, we found the following six protozoa: *Entamoeba histolytica* (*E*. *histolytica*), *Giardia intestinalis* (*G*. *intestinalis*), *Entamoeba coli* (*E*. *coli*), *Endolimax nana* (*E*. *nana*), *Chilomastix mesnili* (*C*. *mesnili*), and *Iodamoeba buetschlii* (*I*. *buetschlii*). *E*. *coli* was the most common protozoan infection. Of the six protozoa detected, *E*. *histolytica*, *E*. *coli*, *E*. *nana*, and *C*. *mesnili* decreased significantly from 2013 to 2015 (P<0.001). The rate of *G*. *intestinalis* infection increased significantly from 16.5% in 2013 to 21.2% in 2015 (P = 0.01). No significant difference was found in the infection rate of *I. buetschlii* during the study period (P = 0.08).

## Discussion

This study revealed changes in intestinal parasitic infection rates after anthelminthic administration to Bolivian children infected with helminths and protozoa. Our procedure was effective for decreasing the overall parasitic infection rate, but we did not see a significant decrease among all the subject parasites during the study period. Of the six helminths detected in this study, the infection rates of *H*. *nana* and *T*. *trichiura* decreased significantly during the period. In the protozoa detected, the rates of *E*. *histolytica*, *E*. *coli*, *E*. *nana*, and *C*. *mesnili* decreased significantly, while *G*. *intestinalis* increased significantly during the study period.

Previous studies have also examined intestinal parasitic infection rates in Bolivians, but results have been inconsistent because of differences in time, area, subjects, and methods. Flores et al. [13] examined 2,723 stool samples collected from 2,521 children and 201 communities in La Paz from 1992 to 1997 using the Kato-Katz method, and the helminth infection rates were 18.0% in children and 23.8% in communities. Tanner et al. [10] examined 92 stool samples collected from children living in lowland Bolivia using the formalin ether-acetate concentration procedure, which detected 70 helminth (76.1%) and 52 protozoan (56.5%) infections. In our previous study conducted in a high altitude area of La Paz in 2012, 274 stool samples collected from La Paz residents, aged 1–71 years, were examined using the medical general laboratory method. Five helminth (1.8%), 107 protozoan (39.1%), and 8 helminth and protozoan (2.9%) infections were detected [14]. The findings obtained in the present study were similar to those in our previous study; helminth infection rate was lower than that of protozoa infection. This difference is thought to be because of unique weather conditions in the study area. Our study areas were located at 3,800 meters (Asis area) and 2,800 meters (Mecapaca area) above sea level, where the annual mean temperature is below 15°C. Generally, helminth eggs are secreted from the body along with humans’ feces, then develop into infective larva under suitable temperature and humidity conditions. A suitable temperature for the development of *Ascaris* eggs is considered to be 15–34°C [29]. Intestinal helminth infections are caused by the ingestion of the infective larva. Helminth eggs take longer time to develop into infective larva when the temperature is less than 15°C. The main reason for the low helminth infection rate in children living in La Paz is because the eggs are unable to develop into infective larva at such low temperatures. Protozoan cysts are resistant forms, remaining infective under low temperature conditions and are infectious causes for protozoan infection. Reduction in the rate of protozoan infections is urgently needed to protect the health of children in this area. Despite the high prevalence of parasitic infections, preventive measures for children in this area have not yet been implemented. Effective and sustainable preventive measures for children in this area are required.

The WHO’s strategy for control of soil-transmitted helminth infection is to prevent the infection and to control morbidity through periodic treatment of at-risk populations living in endemic areas [16]. When our study results for 2013 were checked against the WHO criteria for medication, the treatment was not required because the helminth infection rate was only 10.3%. However, the infection rate for helminths plus pathogenic protozoa was 46.4%, as shown in Table 3. Our results show that treatment with anthelminthic medicine should be administered to children once or twice a year.

Japan previously had a high rate of soil-transmitted helminth infection. Mass stool examination and treatment are some of the factors that successfully decreased the infection rate [16]. We therefore hypothesized that mass stool examination and treatment could also reduce the infection rate in Bolivians. In our study, the overall parasitic infection rate decreased significantly from 65.2% in 2013 to 43.0% in 2015 (22.2 percentage points), following our treatment program (P<0.001). Our data demonstrate the effectiveness of mass stool examination and treatment for decreasing the overall infection rate. However, another interesting finding was the significantly increased infection rate of *G*. *intestinalis* despite anthelminthic administration to infected children. The infection rate of *G*. *intestinalis* was 16.5% in 2013; after anthelminthics were administered, the rate decreased to 7.1% in 2014, but it increased to 21.2% in 2015. A possible reason for this increase is the length of time between anthelminthic administration and stool examination. Although in 2014 stool examination was performed 4–5 months after the treatment, in 2015 it was performed 16–17 months after the treatment. In brief, reinfection, or new infection with *G*. *intestinalis*, appeared in some children between 2014 and 2015. *G*. *intestinalis* infection is caused mainly by the ingestion of cysts in contaminated water, food, or both or by the fecal-oral route through person-to-person transmission [30]. In fact, in 2015, more than 40% of the children in three particular classes in the school were infected with *G*. *intestinalis* (8/20, 40%; 15/34, 44%; 20/32, 63%), suggesting an outbreak in those classes. The CDC recommends “wash the hands with soap and warm water before handling food” as one method for preventing parasitic infection [31]. In the Mecapaca area, many people make their living in agriculture, but the area is always short of usable water. Because the school has no water for washing hands, the school children function under poor environmental sanitation. Therefore, the children are not accustomed to washing their hands before meals. In addition, *Giardia* is known to be a very common intestinal parasite of domestic animals, including livestock, dogs, cats and wildlife [32]. Therefore, people who have contact with these animals may become infected with *Giardia*. However, the risk of humans acquiring *Giardia* infection from these animals is low because the exact type of *Giardia* that infects humans is not usually the same type that infects dogs and cats [33, 34]. We have no data on the frequency of contact between children in our study and animals, however these findings suggest that *Giardia* infection occurred more frequently via contaminated water, food or the fecal-oral route than through direct physical contact with animals. The most important measure to prevent *G*. *intestinalis* infection is good hand hygiene, such as washing hands before handling food. Therefore, it is probable that parasitic infections in the children developed via the fecal-oral route through the absence of hand-washing. The most important method of reducing the risk of infection is to teach children the importance of good hand hygiene. In addition, maintenance and improvement of the water supply is needed to decrease the parasitic infection rate in children.

*T*. *trichiura* infection decreased significantly during the study period. However, the egg was only detected in three children in 2013. There have been no reported cases since 2014, but continued surveillance for this helminth is still needed.

In this study we focused on nitazoxanide for the treatment as an alternative to benzimidazole. This was for a number of reasons: (1) protozoan infection rates were higher than helminth infection rates in the baseline survey in 2013; (2) *H*. *nana* infection was the highest among the helminths we detected in this study; (3) infection rates of soil-transmitted helminths, *A*. *lumbricoides*, *T*. *trichiura*, and hookworm were not as high. Nitazoxanide is an effective treatment for infections caused by protozoa and helminths such as *E*. *histolytica*, *H*. *nana* and *A*. *lumbricoides*, therefore we used this one to decrease the both infection rates. The tablet was administered according to the directions for the children aged 12 years and older. However, syrup was given to the children aged 11 years and younger because it was difficult for younger children to swallow the tablet with water or food. We required two months to treat all the infected children after the stool examinations.

There are some limitations in this study. First, approximately 1,000 children aged 1–19 years attend the school each year. During our study, each year there were new entrants to kindergarten and graduates from high school. Therefore, at the time of the stool examinations, the subjects eligible for the study varied each year. The infection rates obtained might not properly reflect the precise variations, as we were unable to compare the rates obtained from children in the same population. However, we examined the stool samples collected from approximately 60–85% of all the children each year, so we can safely assume that our data reflect the trend of parasitic infection rates among the children. Second, 10% formalin-fixed stool samples were used, so we were unable to find *Blastocystis hominis* in the samples because this protozoa is not easily seen in concentrated wet mount preparations [35]. If this protozoa had been detected, our results might have shown higher protozoan infection rates.

In summary, intestinal parasitic infection rates decreased significantly because of mass stool examination and treatment, but *G*. *intestinalis* infection significantly increased. This increased infection rate may have resulted from the lengthy period between treatment and stool examination. Although our findings need to be compared with the changes in parasitic infection rates after anthelminthic administration every 6 months, they demonstrate the effect of mass stool examination and treatment for decreasing the parasitic infection rates in Bolivian children. There is the potential for a more rapid decrease in parasitic infection rates if the water and sewerage infrastructure is improved in the school and homes in this area.

## Supporting Information

S1 Checklist STROBE Checklist. (DOC)
